# 473. Advancing Autonomy-Supportive Education in Infectious Disease Fellowship: Insights from a Faculty Development Pilot

**DOI:** 10.1093/ofid/ofaf695.161

**Published:** 2026-01-11

**Authors:** Sarah-Ann Keyes, Stacey Rose, Alison Robins

**Affiliations:** Baylor College of Medicine, Houston, Texas; Baylor College of Medicine, Houston, Texas; Baylor College of Medicine, Houston, Texas

## Abstract

**Background:**

Self-determination theory (SDT) posits that autonomy, relatedness, and competency are critical to well-being. SDT is increasingly recognized as relevant to medical education; learning environments that promote autonomy, relatedness and competency benefit both trainees and teachers. However, tools for faculty development in autonomy-supportive teaching are lacking.

**Methods:**

We developed and implemented a faculty development workshop for Infectious Disease (ID) faculty at our institution to help educators engage in autonomy-supportive teaching methods. Faculty participated in a one-hour session with background information on autonomy-supportive teaching, along with interactive cases to practice techniques. A post-session survey was completed by participating faculty to gauge confidence in knowledge and skills gained and intent to apply learned strategies in future interactions with trainees.

**Results:**

Of 20 faculty who participated in the workshop, 12 completed the post-session survey (60%). Following the session, 100% (12/12) of respondents reported being “somewhat familiar” or “very familiar” with SDT concepts, compared to 41.7% (5/12) before the session. Similarly, respondents reported improved confidence in identifying and practicing autonomy-supportive teaching methods: 75% (8/12) self-rated as “very confident” after the session, versus 16.7% (2/12) before the session. On open-ended responses, faculty indicated an intent to incorporate several new strategies, such as “nudging” for learning discovery and emphasizing meaning in work as part of feedback to trainees.

**Conclusion:**

This pilot faculty development workshop appeared effective in improving ID faculty knowledge of SDT and skills for autonomy-supportive teaching. Limitations include the small sample size and potential for respondent bias. The workshop is now being offered to faculty in other disciplines, with customized cases to ensure perceived relevance. Future studies will incorporate trainee input regarding the effect of the workshop on faculty behaviors in the clinical learning environment.

**Disclosures:**

All Authors: No reported disclosures

